# Impacts of keratoconus on quality of life: a qualitative study

**DOI:** 10.1038/s41433-024-03251-6

**Published:** 2024-07-23

**Authors:** Leo Fan, Himal Kandel, Stephanie L. Watson

**Affiliations:** 1 Faculty of Medicine and Health, The University of Sydney, Save Sight Institute, Sydney, NSW Australia; 2 Sydney Eye Hospital, Sydney, NSW Australia

**Keywords:** Corneal diseases, Quality of life

## Abstract

**Background/Aims:**

To identify the domains of quality of life (QoL) in people with keratoconus.

**Methods:**

Semi structured in-depth in person and telephone interviews were conducted with participants diagnosed with keratoconus and recruited from the Sydney Eye Hospital, Sydney, Australia. Thematic analysis of interview content was conducted using inductive and deductive processes. Data was collected until thematic saturation was obtained.

**Results:**

33 patients with keratoconus with median age 37 (range 18 to 65) years and majority male (*n* = 25; 75.8%) were interviewed and a total of 2551 quotes coded. Thematic analysis resulted in 7 broad themes, Driving (199 references), Career (259 references), Symptoms (647 references), Enjoyment (149 references), Relationships (250 references), Financial (104 references) and Healthcare (881 references). Most references described a negative relationship between keratoconus and these 7 domains. The diverse QoL issues expressed included frustration with treatment effectiveness, fear of disease progression, inconvenience with contact lenses, forced career changes and job loss, cost of contact lenses, and feelings of isolation and discrimination. Themes and subthemes described a complex and varied relationship between keratoconus and QoL.

**Conclusion:**

Severe quality of life impairment was experienced by keratoconus patients despite treatment. Keratoconus diminishes various aspects of individual’s QoL. Therapies able to improve QoL are still needed for keratoconus.

## Introduction

Keratoconus is a progressive disorder where central or paracentral thinning of the cornea leads to changes in vision and symptoms of pain and photophobia [1–3]. Visual changes occur from steepening of the cornea which leads to progressive, typically irregular astigmatism [2, 4]. Early treatment is through the prescribing of glasses and/or contact lenses and management of associated risk factors such as atopy, sleep apnoea and eye rubbing [4–6]. Surgery such as cross linking (CXL), intrastromal corneal ring segments (ICRS) or keratoplasty may be indicated [7–9].

Quality of life (QoL) is an important outcome in research in keratoconus treatment and is assessed through standardized questionnaires [10–12]. Keratoconus patients have been shown to score lower on the Impact of Vision Impairment (IVI) questionnaire, which measures activity limitation and emotional impact, when compared to other conditions such as diabetic macular oedema and age-related macular degeneration [13]. The Keratoconus Outcomes Research Questionnaire (KORQ), has content on two domains of QoL (symptoms and activity limitation) and has been externally validated for keratoconus [14].

Current literature reporting qualitative analysis of keratoconus patient experience is sparse, to our knowledge there has been only one recent publication [15] Data was reported in this study on patients receiving a single type of treatment for their keratoconus [15]. Knowledge of the patient experience of keratoconus in everyday practice with management utilizing a range of treatments, is needed to ensure current treatment approaches address patient needs [16] and to inform the development of new questionnaires and treatments.

To address the knowledge gaps on QoL impacts of keratoconus in the real-world, this study aimed to evaluate the patient experience of a broad range of keratoconus patients; that is patients treated in a variety of ways such as by rigid gas permeable contact lens, ICRS, CXL, and/or keratoplasty.

## Methods

Semi-structured phone and face-to-face interviews were conducted with patients diagnosed with keratoconus. Baseline sociodemographic and medical data was obtained through survey and chart review. The study received ethics approval from the Royal Prince Alfred Hospital Research Ethics and Governance Office (Project Number: 2023/ETH00410) and adhered to the principles of the National Statement on Ethical Conduct in Human Research (2023). Standards for reporting qualitative research were followed during the conduct of the study [17].

Inclusion criteria were patients aged >18 years, diagnosed in at least one eye with keratoconus who provided informed consent to participate in an interview. Keratoconus was diagnosed by the treating clinicians utilizing Pentacam topography. Patients were excluded if they had other ocular pathology and comorbidities that investigators considered would affect QoL (e.g., macular degeneration, retinal detachment), or had psychiatric disorders, other medical conditions or cognitive/linguistic limitations making them incapable of participating in an interview in English. Participants were recruited from corneal clinics at the Sydney Eye Hospital, a quaternary referral eye hospital in Sydney, New South Wales, Australia.

Semi-structured interviews lasting approximately 25 min were conducted within 2 weeks of recruitment. Interviews included broad and open-ended questions exploring different aspects of QoL and were conducted in English. Identifiable data was censored in interviews. An interview guide used to assess the experience of refractive error [18] was adapted for this study. Interviews were transcribed verbatim and transcripts were analysed qualitatively using NVivo Software, Version 12 (QSR International Pty Ltd.). Demographic and clinical data collection were collected at time of recruitment from the patient’s medical record.

Phenomenological analysis was performed where discrete concepts were identified through inductive and deductive processes [19]. Discrete concepts which described the relationship between keratoconus and a participant’s QoL were given a code, consisting of a short descriptor of the concept identified. Codes were grouped into categories through their semantic relationship. Categories were grouped into broader themes. Codes, categories, and themes were discussed by investigators on a weekly basis to ensure a consensus view on emerging data [20] Data collection occurred until thematic saturation was reached, that is where new categories of codes were not identified in subsequent interviews.

### Statistical analysis

Descriptive statistics were used for demographic data with analysis in Microsoft Excel.

## Results

### Participants

Thirty-three patients were interviewed of median age was 37 (range 18 to 65) years with most patients male (*n* = 25; 75.8%) and single (*n* = 24; 72.7%). Socio-demographic data and baseline-medical data are reported in Table 1. The most common highest level of educational attainment reported was a Bachelor’s degree (*n* = 11; 33%). Educational attainment ranged from incomplete finishing of high school to a Master’s degree. Patients resided in regions ranging in remoteness from Modified Monash Model categories MM1 (Metropolitan Areas) to MM5 (Small Rural Towns) [21]. There were 7 distinct self-identified racial backgrounds reported, with Caucasian being the most common (*n* = 18; 54.5%).

**Table 1:** Socio-demographic characteristics of patients with keratoconus (*n* = 33).

Characteristics	Total (*n* = 33)
Gender, *n* (%)	
Female	8 (24%)
Male	25 (76%)
Age, years	
Median (range)	37 (18–65)
Relationship status, *n* (%)	
Married	9 (27%)
Single	24 (73%)
Highest level of education, *n* (%)	
Year 10	5 (15%)
Year 12	7 (21%)
Certificate	1 (3%)
Diploma	4 (12%)
Bachelors	11 (33%)
Masters	5 (19%)
Doctorate	0 (0%)
Employment status, *n* (%)	
Employed	31 (94%)
Unemployed	2 (6%)
Rurality, (Modified Monash Model)	
Median (range)	1 (1–5)
Ethnicity, n (%)	
Caucasian	18 (55%)
Asian	6 (18%)
Indian	4 (12%)
Pacific-islander	3 (9%)
Middle Eastern	1 (3%)
African	1 (3%)

Clinical characteristics of the included patients are reported in Table 2. Time since diagnosis of keratoconus had a median duration of 13 (range: 1 to 41) years and the median age at diagnosis was 22 (range 10 to 9) years. All stages of keratoconus, as classified by the Amsler–Krumeich scale, were represented within the patient cohort (33.3% Stage I, 29.6% Stage II, 7.4% Stage III, 29.6% Stage IV) [22]. Habitual visual acuity (logMAR) ranged from 0.0 to 0.80 in the better eye (median 0.2). Most patients reported using corrective devices (spectacles, contact lenses) (*n* = 23; 69.7%) and 10 patients reported not using vision correction devices. Reliance on corrective devices varied from constant use to intermittent. Different modalities of contact lenses (soft, rigid gas permeable, scleral, piggyback) were reported. The history of ophthalmic surgery ranged from surgery naïve (*n* = 13; 39.4%) to multiple surgeries (*n* = 11, 33.3%). Procedures reported by the patients included corneal crosslinking (CXL) (*n* = 8; 24.2%), keratoplasty (*n* = 13; 39.4%), and intrastromal corneal ring segments (ICRS) (*n* = 1; 3.0%).

**Table 2:** Baseline clinical characteristics of patients with keratoconus (*n* = 33).

Characteristics	Total (*n* = 33)
Time since diagnosis, years	
Median (range)	13 (1–41)
Keratoconus Stage (Amsler–Krumeich), *n* (%)^a^	
Stage 1	9 (33%)
Stage 2	8 (30%)
Stage 3	2 (7%)
Stage 4	8 (30%)
Habitual Visual Acuity^b^, logMAR	
Median	0.20
Range	0–0.80
Visual Correction, *n* (%)	
Unaided	10 (30%)
Spectacle	17 (52%)
Contact Lens	6 (18%)
Soft	2 (6%)
Rigid Gas Permeable	3 (9%)
Scleral	2 (6%)
Piggyback	1 (3%)
Surgery, *n* (%)	
No previous surgery	13 (39%)
Multiple surgeries	11 (33%)
Crosslinking	8 (24%)
Keratoplasty	13 (39%)
Intrastromal Corneal Rings	1 (3%)
Other	7 (21%)

^a^Data not available for 6 patients.

^b^Data not available for 3 patients.

### Saturation

**Fig. 1:**
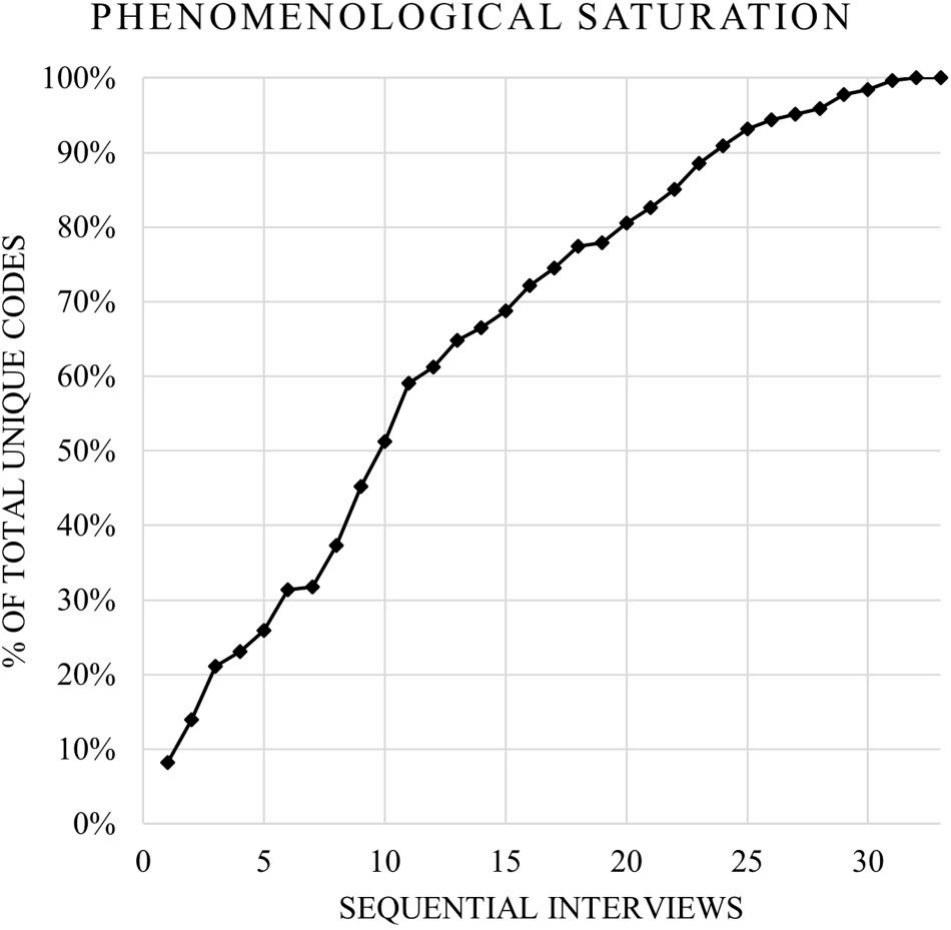
Description: Graph of total unique codes over the duration of the study. 50% of total codes were reached with the first 10 interviews, and saturation was reached with no new codes after the 31st interview.

Phenomenological saturation was reached at 31 participants, with 2 subsequent interviews not revealing new codes. Initial incidence of new phenomena was rapid for the first 10 interviews, where 51% of total unique codes were recorded (Fig. 1).

### Coding

Phenomenological analysis of quotes (references) and codes revealed 7 distinct themes in the relationship between keratoconus and QoL. (Table 3) They are described further with reference to the specific codes and quotes below and summarized in Table 4. A list of all the unique QoL issues identified is available as supplementary material (Appendix 1).

**Table 3:** Identified themes and related codes and references for patients with keratoconus (*n* = 33).

Major Themes	Codes (*n*)	References (*n*)
Healthcare	398	881
Symptoms	218	647
Career	118	259
Enjoyment	92	149
Relationships	75	250
Driving	55	199
Financial	39	104

**Table 4:** Themes and quotes reported by patients with keratoconus (*n* = 33).

Code	Quote (Context – Gender, Age, Treatments)
HEALTHCARE	
H1 – Unprofessional treatment	“The optometrist kept telling me that my prescription was changing every few minutes, and she started getting really annoyed at me. I thought she was very unprofessional” (Male, 40, Glasses)
H2 – Disagreement with doctor over diagnosis	“I had to basically argue with her that I have actually had these scans done before and I’ve had, um, eye professionals, I’m not sure of correct terminology, explain this condition, diagnose this condition, monitor this condition. And yeah, that was just a very negative outcome” (Male, 26, Glasses)
H3 -Appointments have a long waiting time	“The system itself is a little bit frustrating to deal with. Like, we go in there. We wait quite a long time.” (Female, 21)
H4 – Hospital environment is depressing	“I felt very sad and sorry for myself, um, going into the hospital, the, the, the first time. Um, And, you know, “why is this happening to me?” And, and all that. Cause I’d never been to hospital before” (Male 51, Rigid contact lens)
H5 – Fear at initial diagnosis	“, When I first got diagnosed, it did, you know, I was quite scared.” (Male, 65, Glasses, Other surgery)_
H6 – Fear due to not understanding condition	“It scared me a bit. I didn’t know anything about keratoconus.” (Male, 65, Glasses, Other surgery)
H7 – Distraught at initial diagnosis	“I was first diagnosed with keratoconus about, um, about one and a half years ago, and yeah, felt, I felt distraught” (Male, 39, Glasses, Keratoplasty)
H8 -Spectacles do not fully address symptoms	“I also didn’t know what the point of wearing them was because I couldn’t notice any difference in my vision” (Male, 25, Glasses, Keratoplasty)
H9 – Contact lens falls out of eye easily	“They were irritating, I was tearing all the times and my nose was blocked, it was painful in the eye, and they were popping out” (Male, 43, Scleral contact lens)
H10 – CXL is a scary experience	“It was terrifying. Um, I was aware that I was going to be awake during it, but I didn’t realize how scary that was actually going to because I’ve never had any type of surgery before” (Female, 18, Glasses, Crosslinking)
H11 – Keratoplasty post-operative care requirements	“it’s a little bit of a life changing thing with being like sterile. You have to wash your hands, everything, like all the time.” (Male, 23, Glasses, Crosslinking, Keratoplasty)
H12 – Fear of ocular trauma after surgery	“I was very paranoid about anything getting in contact with my eyes, so they didn’t instruct me, but I still went into this habit of just wearing things over my eye,” (Male, 28, Keratoplasty)
H13 – Wish for a cure	“I think the saddest part was that finding out there was no cure. Yeah, it was heartbreaking,” (Male, 37, Keratoplasty, Other surgery)
H14 – Keratoplasty is exciting	“So there wasn’t any anxiety or fear around going into surgery. I was actually kinda excited to be frank to actually have something done about my eye.” (Male, 28, Keratoplasty)
H15 – Keratoplasty improves QoL	“It’s allowed me to get my license… allowed me to have a bit more of a normal life. Having a license and yea I don’t regret it, If I had to go back in time, I’d do it all over again” (Male, 38, Glasses, Crosslinking, Keratoplasty)
H16 – Crosslinking is reassuring	“I don’t really worry about it as much now cuz I do know that I’ve had a cross-linking surgery. I know how it’s, I know that it’s gonna help in the long run.” (Female, 18, Glasses, Crosslinking)
H17 – Glasses improve vision	“Wearing glasses, all of the symptoms kind of just go away and your vision goes back to normal again” (Male, 26, Glasses)
H18- Contact lens improves QoL	“ I can function, I can drive, I can work, I can do most things that I need when I’ve got the lenses on.” (Male, 65, Glasses, Other surgery)
SYMPTOM	
S1 - Blur	“I can’t see it once I come about, say maybe like 10 or 15 meters, then I can see it properly.“ (Male, 52)
S2 – Pain	“Oh, look, even my eyesight became quite blurred and, and, um, And incredibly painful when, well, I wouldn’t say incredibly painful, but quite an-annoyingly painful” (Male, 65, Glasses, Other surgery)
S3 - Headaches	“I can’t be on my phone or technology much, especially when studying, just cuz I get headaches.” (Female, 18, Glasses, Crosslinking)
S4 – Diplopia	“I’ll have like this double vision where the image that I’m looking at, whatever I’m looking at, has like an after image effect above that object.” (Male, 28, Keratoplasty)
S5 – Peripheral vision	“Like you just tend to realize that you kind of. Um, live in, in like a cone. Like you, you’re very dependent, especially me, just with my left eye. I’m very dependent on where my left eye is focusing on.” (Male, 23, Glasses, Crosslinking, Keratoplasty)
S6 – Irritation	“I think later on, the actual eyes started hurting me. Um, It was becoming red and irritated.” (Male, 37, Keratoplasty, Other surgery)
S7 – Difficulty with eye makeup	“if you look close up into a mirror to try and do the eye makeup, it’s so blurry. But then if you’re too far, it’s also too blurry.” (Female, 32, Glasses, Crosslinking, Intrastromal Corneal Ring Segments)
S8 – Self consciousness over red eyes	“I’m extremely self-conscious, even making eye contact with people..” (Female, 35, Rigid lens)
S9 – Futility	“The kind of, the thought that it’s never going to get better sucks, and that’s not great.” (Male, 26, Glasses)
S10 – Inconvenience with coping devices	“You’ve gotta stuff around for 10 or, 5 or 10 minutes, You got to do things like that, Even though I have mobility aids for that sort of thing, you’ve gotta carry that with you. And that’s stuff you’ve got to cart around with you and that becomes a problem” (Male, 62, Glasses, Other surgery)
CAREER	
C1- Falling behind in class	“There’s been many times where I feel like I’m falling behind because I can’t even read the board” (Female, 18, Glasses, Crosslinking)
C2 – Ceasing studies	““I always used to think that, “Oh, if I didn’t have this condition, I would be driving, I would be doing this, I would be doing that.” I wouldn’t probably, you know, have stopped all my studies” (Female, 21)
C3 – Difficulty with whiteboard	“I would find it hard to read what was on the board” (Male, 25)
C4 – Teachers not understanding	““One time I took my phone out to take a photo of this man’s tiny, tiny writing. No one could read it. And he took my phone for almost four days.” (Female, 18, Glasses, Crosslinking)
C5 – Withdrawing from university	“I got to a point where I ended up just withdrawing from uni just because I didn’t feel like I could properly analyze the data to finish off the thesis.” (Female, 32, Glasses, Crosslinking, Intrastromal Corneal Ring Segments)
C6 – Failing medical standards	“I did have an interest back in the day to become a pilot or a helicopter pilot. But, um, with the vision impairment, obviously that can never happen” (Male, 25)
C7- Regret at not pursuing career	“Even now, you know, uh, at 50, I still regret that I didn’t get to do the career I ultimately really wanted to do.” (Male, 49, Glasses, Keratoplasty, Other surgery)
C8 – Career change	“It was a great job. I loved it. But yeah, look, you can’t… What are you gonna complain about? It is kind of one of those things that you just gotta deal with. It’s part of life” (Male, 23, Glasses, Crosslinking, Keratoplasty)
C9 – Fear of career change following treatment	““I suppose with the operation as well I suppose there’s that, uh, the thought of, “oh, do I have to change careers again after this operation?” Uh, you know, downgrade again to something else” (Male, 55, Glasses, Keratoplasty)
C10 – Fired due to keratoconus	” I got fired, got let off, um, because yeah, my, my, my boss at the time was just saying like, “you know, I, I can’t have, uh, you, you are almost like a, a risk. To have onsite. Um, it’s dangerous.” (Male, 23, Glasses, Crosslinking, Keratoplasty)
C11 – Limited career progression	“I’ve done a lot of lateral transfers at work as opposed to moving up the chain just because I feel like with more responsibility, particularly at my company, like as you go up, you have a larger and larger budget to manage. And it’s those numbers and dealing with numbers that I worry about.” (Female, 32, Glasses, Crosslinking, Intrastromal Corneal Ring Segments)
C12 – Feeling incompetent at work	“Anyone else or anything else you would’ve thought “God easy-peasy”, you know, Cause you couldn’t see, you think you get frustrated, you get, um, annoyed. You feel incompetent.” (Male, 55, Glasses, Keratoplasty)
C13 – Moving closer to screens	“If I have to use a computer, my face is basically plastered up against the screen. And, um, I hate that because it looks stupid..” (Male, 28, Keratoplasty)
C14 – Self conscious at work	“I can’t have the screen too close to me in the office. Cause people probably think I’m really, really blind.” (Female 30, Glasses)
ENJOYMENT	
E1 – Stopping hobbies	“I used to like to do puzzles. I can’t do puzzles that much anymore. I stopped buying them.” (Male, 52)
E2 – Missing out on experiencing life	“I’ve been like living under a rock, like just seeing things and not the best kind of like light. And I’m also an artist. I like to draw and I like to paint and like, I just feel like I’ve been like left out.” (Female 30, Glasses)
E3- Cannot recognize teammates	“I will constantly pass the ball to the wrong person, the wrong team, and I simply cannot identify the players by their face or their, their overall stature or whatever.” (Male, 26, Glasses)
E4 – Limited confidence	“I used to be very good. At, at FPS games. And now I struggle, I struggle so much that in the last eight to nine months I haven’t jumped on. Cause I just don’t feel confident. Like the desire’s gone.” (Male, 50, Glasses)
E5 – Loss of passion for sport	“I was getting worse and worse, I just fell outta love and I just stopped doing it as well.” (Male, 23, Glasses, Crosslinking, Keratoplasty)
E6- Sadness at stopping hobbies	“I feel a bit up upset, but what can I do about it?You know?. Yeah. I can’t do nothing about it.” (Male, 52)
E7 – Discomfort during air flights due to contact lens	“catching flights is very difficult. The air on the flight, it’s very dry. I need to take the lens out, but then I also need to make sure that I time my, um, I make sure, I have to make sure the lens is back in” (Female, 35, Rigid lens)
E8 – Sadness at not being able to see kids succeed	“look, definitely upset me in relation to, uh, not being able to see my kids, uh, work clearly when they’re, they’re doing their things. That, that has impacted me um, terribly,” (Male, 49, Glasses, Keratoplasty, Other surgery)
E9 – Missing visual element of enjoyment	“It doesn’t do justice. Like if I go out and like I’m sight-seeing, so I was in Korea like a few months ago and I’m like, “it’s really pretty”. But like I would know that had I had perfect vision, I would, I’d be able to capture it better” (Female 30, Glasses)
E10 – Difficulty reading menu at restaurant	“when I go out with my friends, I cannot look at the menu to order anything.” (Female, 23)
E11 – Cannot read sheet music	“I used to play guitar quite a lot and I’d read off sheet paper, but now I can’t even look at sheet paper anymore, like right in front of my face cuz it’s just so blurry and like I’ve given up that the hobby” (Female, 18, Glasses, Crosslinking)
RELATIONSHIPS	
R1 – Isolation	“sometimes it would drain your motivation to go out and go to an event. Like could be a little thing like a birthday party or your friends invited you out, or maybe even a sport game or whatnot. You kind of just wanna, you know, lay down and close your eyes and not having to worry about anything.” (Male, 25)
R2 – Lack of Understanding	“I didn’t have my license, so they were like, “oh, when are you gonna get it, when are you gonna get it, your eyes are fine” (Male, 38, Glasses, Crosslinking, Keratoplasty)
R3 – Avoiding relationships	“Like I think initially, like, you know, I mentioned that I felt like it was a disability for me. So for a long time I avoided like, intimate relationships.” (Female, 32, Glasses, Crosslinking, Intrastromal Corneal Ring Segments)
R4 – Not developing friendships	“I’ve only been there for two years and I have some people who really want to be a friend and have drinks and do things outside of work, and I just can’t be bothered explaining my health history.” (Female, 35, Rigid lens)
R5 – Being seen as different	“If I have my laptop and I’m out having coffee, but I’m still working like, and I don’t have my glasses, people probably think I’m weird because I’m squinting and like struggling..” (Female 30, Glasses)
R6 – Self-conscious over appearance	“When someone looks at you closer into the eyes, they can see that round bit that’s been transplanted. I’ve had that a lot in the past.” (Male, 55, Glasses, Keratoplasty)
R7 – Experiencing discrimination	“She told me that, “oh, like, so you’re disabled?”. So I’ve been like, I felt discriminated many times.” (Female, 23)
R8 – Awkward interactions at work	“I, virtually until I’m up right in front of you, I will not be able to see your face. That also makes it very awkward in a lot of situations” (Male, 23, Glasses, Crosslinking, Keratoplasty)
R9 – Not discussing condition	“I don’t talk about it to no one. I have to be honest with you. Like, this is me personally though. Um, I don’t talk about it to no one. So, uh, I just keep it to myself, you know.” (Male, 37, Keratoplasty, Other surgery)
R10 – Conflict with partner	“And you know, my missus, she blows up about it all the time. I’ll be doing the dishes. And we, we hand clean all of our dishes and there’ll still be like little bits of like, you know, food and stuff that I just didn’t see” (Male, 23, Glasses, Crosslinking, Keratoplasty)
R11 – Feelings of guilt due to relying on partner	“I kind of feel bad in that sense that okay, let’s say if we wanna go somewhere, my partner takes most of the bill. Like I try and chip in as much as I can, but it’s really hard” (Female, 23)
R12 - Support from family	“I really am fortunate, uh, for my family’s understanding. As well as my social life’s understanding, uh, because they understand” (Participant 35)
DRIVING	
D1 – Conditions; Rain	“If we have like really rainy, dark cold day, then I’m not gonna go. Cause I don’t wanna drive.” (Female, 21)
D2 – Conditions; Night	“So during the day my vision is okay, but, um, trying to see at nighttime, it’s troubling and, um, I can’t see the road as well.” (Male, 39, Glasses, Keratoplasty)
D3 – Discomfort of driving	“I’m borderline. Like technically legal. Legally I can, um, but I, I would not feel comfortable with driving” (Male, 23, Glasses, Crosslinking, Keratoplasty)
D4 – Importance of driving	“But there’s other jobs that I’ve, I’ve, I’ve considered that I need to drive constantly and if I can’t drive, I can’t pursue those, those prospects.” (Male, 57, Glasses, Keratoplasty)
D5 – Relying on others to drive	“I can’t remember instances where I’ve missed out on things, but that’s mainly because of like my friends, and I think this is probably additionally impacts them, is because they’re the ones that have to drive at night because I’m not confident to do it.” (Female, 21)
D6 – Annoyance at blurred vision	“It’s just, it’s, it’s not that it’s changing my driving, almost nothing, but it’s just, it’s just annoying. I still can see and I still can distinguish, it’s not that I… my vision is that poor. If I couldn’t see, I wouldn’t drive, but it’s just annoying.” (Male, 43, Scleral contact lens)
D7 – Fear of crashing	“I can’t see or I’ll miss something. And that, that piece just terrifies me..it just terrifies me.,” (Male 51, Rigid contact lens,)
D8 – Needing sunglasses to drive	“if I don’t have those glasses there, it can at times make it impossible for me to, to be able see, or be able to drive.” (Male 51, Rigid contact lens,)
D9 – Difficulty with other lights	“the amount of like haloes and stretch lights from oncoming cars and traffic lights was just ridiculous really. And there’s blurry lights covering the road” (Male, 26, Glasses)
D10 – Difficulty Parking	“If I had to go into a shopping center, I wouldn’t be able to, like, I just didn’t feel confident reversing into a spot” (Female, 32, Glasses, Crosslinking, Intrastromal Corneal Ring Segments)
D11 – Fear of losing driver’s license	“That next fear is, you know, if my vision’s not good enough, I won’t get my driver’s license next.” (Male 51, Rigid contact lens,)
D12 – Concerns and issues with driving license	“I was worried, with having the vision issues that I wouldn’t be able to pass any tests and be actually able to get the license if I tried again.” (Male, 27)
D13 – Difficulty seeing obstacles	“Its just harder to see obstacles, especially at night time.” (Male, 39, Glasses, Keratoplasty)
D14 – Discomfort driving	“There’s no way that I would. Um, be able to drive confidently knowing where I’m going and what speed I’m meant to be going. Like even looking at the speedometer in the car, I’ll be squinting and spending a lot of time finding out where I’m sitting on the speedometer. I’m not really looking at the road.” (Male, 23, Glasses, Crosslinking, Keratoplasty)
D15 – Driving slowly	“I’ll have to put on the, high beams And, um, go slow, in especially an area, in a normal road that, I don’t know.” (Male, 39, Glasses, Keratoplasty)
D16 – Avoiding highways	“I try not to take the highways as often. I try to take, uh, like smaller, like back streets, roads.” (Male, 24, Glasses, Crosslinking)
D17 – Public transport is unreliable	“The biggest problem is mobility, you’ve got to rely on public transport. You’ve gotta rely on unreliable public transport.” (Male, 62, Glasses, Other surgery)
FINANCIAL	
F1 – Rigid lens; Cost of lens is high	“Well financial aspect is… one lens, it costs like at least one and a half thousand dollars. So, yeah, it’s a burden.” (Male, 43, Scleral contact lens)
F2 – Direct costs; Treatment; Surgery	“I went, saw a specialist. And he wanted all the money front, which we couldn’t afford at the time. He wanted, uh, close to $11,000 upfront to do the operation.” (Male, 55, Glasses, Keratoplasty)
F3 – Ongoing cost of solutions	“The solutions are. Um, expensive. Um, not extremely expensive. However, there is an expiry date on the solutions. For example, I use Boston Eye Conditioning Solution. Um, this costs about $16 and I need to purchase this every month along with saline solution and the cleaning, uh, a Boston cleaning, uh, bleach.” (Female, 35, Rigid lens)
F4 – Multiple pairs of glasses are expensive	“Purchasing glasses, that’s been pretty expensive. I mean, a, a decent script in frames is what, six, 700 bucks? And I’ve had to buy four pairs of glasses, so, That’s not great” (Male, 26, Glasses)
F5 – Needing to save for treatment	“It took us months and months to save up that money. Um, you know, I had a job at the time, I was working at 14, 15 at a fish and chip store. I was contributing as much as I could” (Male, 23, Glasses, Crosslinking, Keratoplasty)
F6 – Ongoing cost of updating contact lens	“It’s almost wasting your money because every six months you’re gonna have to buy new hard contact lenses and your eyes getting worse and worse, and you’re gonna change, and it’s just gonna be very expensive” (Male, 23, Glasses, Crosslinking, Keratoplasty)
F7 – Cost of treatment for injuries associated with keratoconus	“”Well, uh, apart from medical bills, costs and things like that, I don’t think its affected me that much. Perhaps the cost of accidents you get into, so, you know, For example, my tooth has cost $10,000 to get fixed. You know? You know, can I contribute that to keratoconus or just sheer stupidity and clumsiness? I don’t know.” (Male, 65, Glasses, Other surgery)
F8 – Indirect cost of travel for treatment	“we’d constantly have to make the flight six hour trip down to, to, to see the, the doc, the doctors. Uh, the, um, the, the surgeon that did it so he would monitor it and um, you know, it was quite costly and everything like that.” (Male, 23, Glasses, Crosslinking, Keratoplasty)
F9 – Family financial strain	“It’s definitely put a strain on my family with the money. Like we come from pretty poor family,” (Male, 23, Glasses, Crosslinking, Keratoplasty)
F10 – Rigid lens expensive relative to student income	“I think it was like 250 per eye. And back then when I was just like a student and like doing part-time work, it was a lot. And I’m not on a health fund too.” (Female 30, Glasses)
F11 – Difficulty accessing welfare	“It’s, yeah, it is frustrating cause like the government wouldn’t even care. Like I’m not even classed as disabled because like they would tell me that, you know, your condition can be fixed” (Female,37, Rigid lens)
F12 – Income limited by job opportunities	“It’s really hard for me to find a job. And even if I do find a job, the job wouldn’t pay me as much as, you know, I would like to earn.” (Female, 23)
OTHER	
O1 – Reliance on less affected eye	“Um, driving at night was a bit different. I never had any difficulties per se cause I still had my left eye, which could see clearly, it still can. So, um, yeah, driving was okay.” (Male, 28, Keratoplasty)
O2 – Acceptance of condition	“Pretty much everything. Um, it is something that you just have to accept early on.” (Male, 29, Scleral lens, Crosslinking, Other surgery)
O3 – Patient expresses minimal impact on QoL	“Uh, it doesn’t really affect me too much. Like there are, there are times, um, I guess it’s not nice as well when you kind of cover your left eye and then you kind of see yourself that its blurry, I guess that’s maybe sometimes a worry, like, “ok, is it gonna get worse?” and everything like that.” (Male, 39)

### Theme 1: Healthcare

Several patients reported negative experiences with availability and quality of treatment; issues included poor access and conflict with practitioners (Table 4, H1,H2). Appointments were frustrating and patient’s first experience with a depressing hospital setting (Table 4, H3,H4). Diagnosis had emotional effects, with recounts of futility, fear, and dismay (Table 4, H5,H6,H7). Treatments, both medical and surgical, were described as limited in effectiveness, and the side effects and recovery were a burden. (Table 4, H8, H9). Personal image was affected by treatment. Contact lenses were described as inconvenient, fragile, and frequently fell out of patient’s eyes (Table 4, H9). Overall, there was expressed frustration with treatment.

Surgery was described as scary and had a challenging recovery period (Table 4, H10, H11). Fears and inconveniences were described during and after surgical treatment (Table 4, H12). Multiple patients expressed their fears when having surgery without general anaesthesia. Patient’s expressed sadness at a perceived lack of cure for keratoconus. (Table 4, H13)

While many negative aspects of treatment were described, patients also described positive aspects, such as excitement at undergoing keratoplasty and improvement in their QoL afterwards (Table 4, H14, H15). Crosslinking provided reassurance to the patients. (Table 4, H16). Both contact lenses and glasses improved vision and QoL (Table 4, H17, H18).

### Theme 2: Symptom

Patients described a direct and indirect burden of symptoms. Symptoms were both acute and chronic and included blurred vision, pain, headaches, dryness, irritation, starburst patterns of light, photophobia, watery eyes, sensitivity, poor depth perception, diplopia, loss of peripheral vision, and swollen, red eyes (Table 4, S1, S2, S3, S4, S5, S6). These symptoms occurred in combinations and during acute episodes (infections, hydrops, and graft rejection) as well as chronically.

Visual symptoms inconvenienced patients and impacted daily activities such as computer usage, reading, and self-care routines (Table 4, S7). Non-visual symptoms caused physical discomfort and self-consciousness due to the appearance of their eyes (Table 4, S8). Patients were emotionally impacted due to these symptoms and described annoyance and futility (Table 4, S9). Symptoms were noticed before a formal diagnosis and were often ignored by the patient. Coping mechanisms to avoid symptoms were described by the patients (Table 4, S10).

### Theme 3: Career

Impacts on the participants education and early working life were recounted. Education was affected early on in life; patients reported being disengaged as students (Table 4, C1, C2). Schoolwork was affected due to visual symptoms which reduced class participation (Table 4, C3) and support from teachers was limited. (Table 4, C4). Delayed recovery following surgery and associated symptoms were described as causing withdrawal from studies (Table 4, C5). Multiple participants described an inability (both perceived and experienced) to meet vision standards for their ideal career (Table 4, C6); these effects caused frustration, sadness, and regret (Table 4, C7).

Early career opportunities were also reported as limited due to issues with education, perceived liability as well as limited independence (Table 4, C2). Keratoconus patients recounted forced career changes as their condition progressed and their aptitude at work decreased (Table 4, C8, C9). Career progression was affected; patients were terminated from their employment due to their condition or felt unable to seek a promotion (Table 4, C10, C11).

Screen usage and tasks requiring fine detail were two salient aspects of work that were recounted as difficult. Patients described errors they had made with their work and resultant feelings of frustration and incompetence (Table 4, C12). Coping mechanisms included multiple monitors, enlarged font, higher contrast screens, magnification devices and support from coworkers (Table 4, C13). Patients were embarrassed by needing to utilize such coping mechanisms (Table 4, C14).

### Theme 4: Enjoyment

A reduced ability to enjoy life was reported. Direct symptoms of the condition or indirect effects due to treatment reduced the patients’ ability to participate in, perform and enjoy pursuits. Activities affected included going to concerts, playing sports, swimming, gaming, and creative pursuits (Table 4, E1). Participants wished for better sight to enable them to enjoy such activities and experiences (Table 4, E2). Sports were mentioned often, with patients reported limited participation in sports and issues with glasses or contact lenses during sporting activities (Table 4, E3).

Patients lost confidence in competitive hobbies (Table 4, E4) and lost passion in their interests (Table 4 E5) had emotional impacts on patients (Table 4, E6). Travel was restricted due to the constraints of hospital appointments. Patients were under stress due to being reliant on treatments (Table 4, E7). Visual enjoyment of experiences such as sight-seeing, attending concerts, or family time was reduced (Table 4, E8, E9, E10). Reading was another issue which affected the enjoyment of movies, musicianship, device usage and television (Table 4, E11).

### Theme 5: Relationships

Keratoconus patients described impacts on their relationships with family, friends, and romantic partners. Patients felt poorly understood and isolated (Table 4, R1, R2); isolation occurred due to symptom burden, an inability to socialize and self-consciousness (Table 4, R3,R4). There were image concerns due to symptoms as well as due to the appearance of corneal grafts (Table 4, R5, R6). These concerns were reinforced by discrimination in different social environments (Table 4, R7). Patients expressed wishes to appear ‘normal’ at an early age, particularly in school and work environments. Difficulty recognizing others led to awkward social situations (Table 4, R8). Participants did not to discuss their condition as they worried it would bore or burden others (Table 4, R9). Conflict occurred in social settings due to feelings of frustration, poor ability perceived by colleagues and burden placed on others (Table 4, R10). Patients expressed both feelings of guilt and shame from burdening others as well as appreciation for support from friends and family (Table 4, R11, R12).

### Theme 6: Driving

Many patients reported impacts on driving due to poor vision, especially when driving at night, in the rain, or in unfamiliar places (Table 4, D1,D2). This affected the patients’ confidence, driving ability, and their overall life, including relationships, career, and hobbies (Table 4, D3,D4,D5). Coping mechanisms and negative emotions that related to driving were also prevalent (Table 4, D6, D7). Challenges included difficulty reading signs, glare from headlights, and reliance on visual aids (Table 4, D8, D9). Car accidents and fear of crashing were common concerns. Surgery recovery worsened vision and impacted driving safety. Depth perception issues made parking challenging (Table 4, D10).

Acquiring a license, meeting vision standards, and fears of license loss were recurrent sub-themes (Table 4, D11, D12). Discomfort, poor object recognition, and obscured vision characterized driving (Table 4, D13, D14). Shame and judgment were associated with the inability to drive. Driving was reported as crucial for careers and social life. Emotions associated with driving included fear, discomfort, annoyance, and sadness. Concerns for safety led to driving slower or alternative routes (Table 4, D15, D16). Patients reported relying on public transport which was inefficient and unreliable (Table 4, D17).

### Theme 7: Financial

Participants described both direct and indirect costs associated with keratoconus. Large one-off financial costs were associated with surgical or contact lens treatment, and smaller ongoing costs with the chronic nature of the condition (Table 4, F1, F2). Ongoing costs included replacement of contact lens solution, medications, nutritional supplements, and visual aids (Table 4, F3, F4). Direct costs were described as expensive and sometimes delayed treatment (Table 4, F5). High-cost items such as glasses and rigid contact lenses were frequently replaced due to damage, loss, or reduced efficacy (Table 4, F4, F6). Indirect costs included treatment due to injuries sustained from poor vision, increased insurance premiums or payment for services to assist with activities of daily living (Table 4, F7, F8).

Due to the early onset of the condition, costs were borne not just by patients, but by their families leading to feelings of guilt, shame or worries of being a burden (Table 4, F9). The younger age at which costs occurred made treatment less affordable relative to patient income (Table 4, F10). The career impacts of keratoconus also limited potential income, and participants described difficulty accessing financial aid. (Table 4, F11, F12)

### Other findings

Some phenomena described by patients did not fit under the broad themes described above. Multiple participants reported a reliance on their less-affected eye (Table 4, O1). For multiple participants, the unilateral or asymmetric presentation of their condition resulted in QoL being minimally affected. For most patients interviewed, there was a reported acceptance of the condition and overall emotional impact of their experience. (Table 4, O2, O3).

## Discussion

In keratoconus, multiple impacts on a patient’s QoL were found. Healthcare experiences were marked by perceived inefficiency and emotional strain. Symptoms, both visual and non-visual, impeded daily life and required coping mechanisms. Education and careers were affected which limited opportunities in life. Enjoyment of life was limited by the condition. Socially, patients felt isolated, misunderstood, and guilty. Limitations on the ability to drive and financial burdens of the condition reduced independence. The varied codes that arose from phenomenological analysis characterized the complexity between the relationship between keratoconus and QoL.

Kennedy et al described keratoconus as having an impact disproportionate to its clinical severity due to the young age at which patients were affected [23]. Similarly, our patients described being affected at a critical stage of their lives when they were unable to bear the financial costs of the condition and its treatment, were beginning their careers and romantic relationships and had no prior experience with surgery. The early onset, chronic and progressive nature of keratoconus as a cause for reduction in QoL in multiple domains has been described [13, 24]. This was also reflected in our results, where patients described early impacts, chronic symptom burden and fear of progression. In ocular conditions such as macular degeneration, patients have described difficulties with aspects of QoL such as self-care; this was not a prominent theme in our interviewed keratoconus patients [25]. In contrast, qualitative research into nystagmus has shown patient concerns with self-image, which was also present in our study [26]. Of interest was the findings in relation to screen-use, as they are becoming an increasing part of modern society, with our patients having difficulty using screens and taking measures such as increasing font size, higher contrast, and larger monitors.

QoL questionnaire scoring has been shown to not always correlate with clinical measurements such as visual acuity [25, 27, 28]. This relationship was reflected qualitatively in our findings, where patients described reduced QoL despite good visual acuity. Tan et al demonstrated a stronger correlation between reading and mobility scores with best corrected visual acuity (BCVA) in the better eye whereas emotional scores correlated more strongly with the BCVA in the worse eye [27]. This complex relationship between vision and QoL effects was reflected in interview responses in our study. Emotional impacts included fear, anxiety and stress due to the healthcare experiences which related to the treated eyes in this study, while a proportion of participants with unilateral presentations of keratoconus described minimal effects on QoL due to reliance on their better eye.

The identified domains of QoL affected in our keratoconus patients will inform the future creation of robust and comprehensive keratoconus specific QoL measures. Questionnaires such as the National Eye Institute 25-Item Visual Function Questionnaire and IVI have been found to be unable to differentiate clinical subgroups of keratoconus [29–31]. While questionnaires specific to keratoconus currently exist such as the KORQ and the Keratoconus End-Points Assessment Questionnaire and Keratoconus Symptom and Severity Questionnaire [32], only the KORQ has been externally validated using modern psychometric methods [14]. The KORQ is also limited to two specific domains of QoL, and does not assess psychosocial impacts [14]. This research has investigated psychosocial elements of keratoconus and described specific effects in different contexts; these data may be utilized to update the KORQ.

There were limitations to this study. Recruitment was from clinics of a public eye hospital, this excluded undiagnosed patients or those seeing private ophthalmologists. This may have contributed to an overrepresentation of experiences related to surgery. The exclusion of patients with other ocular conditions also excluded patients who developed glaucoma and/or cataracts secondary to topical steroid usage following keratoplasty for keratoconus. The qualitative nature of this study may have led to bias due to preconceptions of the researchers. Guidelines for qualitative research by Malterud et al were followed to minimize this bias [20]. Furthermore, open ended questions were used, patients were assured of the anonymity of their responses and self-reflection and feedback with other investigators weekly was employed to reduce inherent bias. Most experiences reported in this study were negative, this may be due to the lack of cure for keratoconus and should not undermine the value of treatments and healthcare to improve patient QoL. In a quantitative study using the KORQ, corneal cross-linking was associated with a significant improvement in most areas of visual functioning and symptoms [33].

In conclusion, keratoconus patient’s lives were impacted significantly with affects across multiple domains. Patients had concerns about their healthcare, symptoms, as well as the psychosocial and economic burden from keratoconus. Future tools which measure QoL in keratoconus should consider these factors.

## Summary

### What was known before

- Keratoconus is associated with poorer quality of life scores on standardized questionnaires compared to other conditions. Affected quality of life domains have been identified in keratoconus patients with single treatment modalities.

### What this study adds

- This study has identified unique quality of life domains impacted in patients with none, single or multiple treatment modalities. Identified domains can contribute to the development of robust keratoconus-specific quality-of-life questionnaires, improve clinician understanding of patient’s experiences and inform the development of new treatments.

## Supplementary information

Appendix 1

## Data Availability

The datasets generated during and/or analysed during the current study are not publicly available but are available from the corresponding author on reasonable request.

## References

[1] Kanski JJ. Clinical ophthalmology: a systematic approach. 4th ed. Oxford; Boston: Butterworth-Heinemann; 1999.

[2] Ferdi A, Nguyen V, Gore D, Allan B, Rozena J, Watson S. Keratoconus natural progression: a systematic review and meta-analysis of 11,529 eyes. Ophthalmology. 2019;126:935–45. 30858022 10.1016/j.ophtha.2019.02.029

[3] Rabinowitz YS. Keratoconus. Surv Ophthalmol. 1998;42:297–319. 9493273 10.1016/s0039-6257(97)00119-7

[4] Tuft SJ, Moodaley LC, Gregory WM, Davison CR, Buckley RJ. Prognostic factors for the progression of keratoconus. Ophthalmology. 1994;101:439–47. 8127564 10.1016/s0161-6420(94)31313-3

[5] Bawazeer AM, Hodge WG, Lorimer B. Atopy and keratoconus: a multivariate analysis. Br J Ophthalmol. 2000;84:834. 10.1136/bjo.84.8.834 PMC1723585 10906086

[6] de Azevedo Magalhaes O, Goncalves MC, Gatinel D. The role of environment in the pathogenesis of keratoconus. Curr Opin Ophthalmol. 2021;32:379–84. 33966012 10.1097/ICU.0000000000000764

[7] Kandel H, Abbondanza M, Gupta A, Mills R, Watson AS, Petsoglou C, et al. Comparison of standard versus accelerated corneal collagen cross-linking for keratoconus: 5-year outcomes from the Save Sight Keratoconus Registry. Eye. 2024;38:95–102. 10.1038/s41433-023-02641-6 PMC10764350 37369766

[8] Belin M, Lim L, Rajpal R, Hafezi F, Gomes J, Cochener B. Corneal cross-linking current USA Status: report from the Cornea Society. Cornea. 2018;37:1218–25. 30067537 10.1097/ICO.0000000000001707

[9] United States Multicenter Clinical Trial of Corneal Collagen Crosslinking for Keratoconus Treatment. 2017. 10.1016/j.ophtha.2017.03.052 28495149

[10] Larkin DFP, Chowdhury K, Burr JM, Raynor M, Edwards M, Tuft SJ, et al. Effect of corneal cross-linking versus standard care on keratoconus progression in young patients: the KERALINK Randomized Controlled Trial. Ophthalmology. 2021;128:1516–26. 33892046 10.1016/j.ophtha.2021.04.019

[11] Wagner H, Barr JT, Zadnik K. Collaborative longitudinal evaluation of keratoconus (CLEK) study: methods and findings to date. Cont Lens Anterior Eye. 2007;30:223–32. 17481941 10.1016/j.clae.2007.03.001 PMC3966142

[12] van den Biggelaar FJ, Cheng YY, Nuijts RM, Schouten JS, Wijdh RJ, Pels E, et al. Economic evaluation of deep anterior lamellar keratoplasty versus penetrating keratoplasty in The Netherlands. Am J Ophthalmol. 2011;151:449–59.e2. 21236411 10.1016/j.ajo.2010.09.012

[13] Kandel H, Nguyen V, Piermarocchi S, Ceklic L, Teo K, Arnalich-Montiel F, et al. Quality of life impact of eye diseases: a Save Sight Registries study. Clin Exp Ophthalmol. 2022;50:386–97. 35080803 10.1111/ceo.14050 PMC9303885

[14] Kandel H, Pesudovs K, Ferdi A, Mills R, Chen JY, Watson A, et al. Psychometric properties of the keratoconus outcomes research questionnaire: a save sight keratoconus registry study. Cornea. 2020;39:303–10. 31634230 10.1097/ICO.0000000000002169

[15] Fournie P, Acquadro M, Touboul D, Cochener B, Chiambaretta F, Muraine M, et al. Keratoconus and the impact of treatment on patients’ quality of life: a qualitative study. Ophthalmol Ther. 2023;08:08. 10.1007/s40123-023-00717-w PMC10287584 37157013

[16] Basch E. Patient-reported outcomes—harnessing patients’ voices to improve clinical care. N Engl J Med. 2017;376:105–8. 28076708 10.1056/NEJMp1611252

[17] O’Brien BC, Harris IB, Beckman TJ, Reed DA, Cook DA. Standards for reporting qualitative research: a synthesis of recommendations. Acad Med. 2014;89:1245–51. 24979285 10.1097/ACM.0000000000000388

[18] Kandel H, Khadka J, Shrestha MK, Sharma S, Neupane Kandel S, Dhungana P, et al. Uncorrected and corrected refractive error experiences of Nepalese adults: a qualitative study. Ophthalmic Epidemiol. 2018;25:147–61. 28985110 10.1080/09286586.2017.1376338

[19] Fereday J, Muir-Cochrane E. Demonstrating rigor using thematic analysis: a hybrid approach of inductive and deductive coding and theme development. Int J Qual Methods. 2006;5:80–92.

[20] Malterud K. Qualitative research: standards, challenges, and guidelines. Lancet. 2001;358:483–8. 11513933 10.1016/S0140-6736(01)05627-6

[21] Versace VL, Skinner TC, Bourke L, Harvey P, Barnett T. National analysis of the Modified Monash Model, population distribution and a socio-economic index to inform rural health workforce planning. Aust J Rural Health. 2021;29:801–10. 34672057 10.1111/ajr.12805

[22] Kamiya K, Ishii R, Shimizu K, Igarashi A. Evaluation of corneal elevation, pachymetry and keratometry in keratoconic eyes with respect to the stage of Amsler-Krumeich classification. Br J Ophthalmol. 2014;98:459–63. 24457362 10.1136/bjophthalmol-2013-304132

[23] Kennedy RH, Bourne WM, Dyer JA. A 48-year clinical and epidemiologic study of keratoconus. Am J Ophthalmol. 1986;101:267–73. 3513592 10.1016/0002-9394(86)90817-2

[24] Pinto RDP, Abe RY, Gomes FC, Barbisan PRT, Martini AF, de Almeida Borges D, et al. Quality of life in keratoconus: evaluation with Keratoconus Outcomes Research Questionnaire (KORQ). Sci. 2021;11:12970. 10.1038/s41598-021-92346-1 PMC8217221 34155238

[25] Hassell JB, Lamoureux EL, Keeffe JE. Impact of age related macular degeneration on quality of life. Br J Ophthalmol. 2006;90:593–6. 16622089 10.1136/bjo.2005.086595 PMC1857044

[26] McLean RJ, Windridge KC, Gottlob I. Living with nystagmus: a qualitative study. Br J Ophthalmol. 2012;96:981–6. 22517800 10.1136/bjophthalmol-2011-301183

[27] Tan J, Ferdi A, Nguyen V, Dinh A, Herrera-Bond A, Watson S. Vision-related quality of life in keratoconus. Clin Exp Ophthalmol. 2017;45:76.

[28] Yeung SN, Lichtinger A, Kim P, Amiran MD, Rootman DS. Retrospective contralateral study comparing deep anterior lamellar keratoplasty with penetrating keratoplasty: a patient’s perspective. Can J Ophthalmol. 2012;47:360–4. 22883846 10.1016/j.jcjo.2012.04.002

[29] Kandel H, Pesudovs K, Watson SL. Measurement of quality of life in keratoconus. Cornea. 2020;39:386–93. 31599780 10.1097/ICO.0000000000002170

[30] Kandel H, Pesudovs K, Nguyen V, Chen JY, Poon A, Mills R, et al. Patient-reported outcomes in keratoconus: a save sight keratoconus registry study. Cornea. 2023;42:590–7. 36036705 10.1097/ICO.0000000000003119

[31] Aydin Kurna S, Altun A, Gencaga T, Akkaya S, Sengor T. Vision related quality of life in patients with keratoconus. J Ophthalmol. 2014;2014:694542. 24868455 10.1155/2014/694542 PMC4020211

[32] Balparda K, Herrera-Chalarca T, Silva-Quintero LA, Torres-Soto SA, Vanegas-Ramirez CM. Development and validation of the “Keratoconus End-Points Assessment Questionnaire” (KEPAQ), a disease-specific instrument for evaluating subjective emotional distress and visual function through Rasch analysis. Clin Ophthalmol. 2020;14:1287–96. 32494119 10.2147/OPTH.S254370 PMC7229796

[33] Kandel H, Chen JY, Sahebjada S, Chong EW, Wiffen S, Watson SL. Cross-linking improves the quality of life of people with keratoconus: a cross-sectional and longitudinal study from the save sight keratoconus registry. Cornea. 2022;13:13. 10.1097/ICO.0000000000003185 36729643

