# Supplementary material for: Impacts of keratoconus on quality of life: a qualitative study
Source: Eye (Lond). 2024 Jul 23;38(16):3136–44. doi: 10.1038/s41433-024-03251-6 (PMC11544024; doi:10.1038/s41433-024-03251-6)
Supplement: Supplementary file 1 — Appendix 1 [file 41433_2024_3251_MOESM1_ESM.docx]

**Supplementary Material:**

**Appendix 1: List of unique issues identified for each domain.**

**Theme I: Healthcare**

Apathy towards stable condition ,Delayed seeking treatment due to minimal impact of condition ,Diagnosis makes patient feel distraught ,Disappointment at lack of treatment options ,Experiencing the worst case scenario ,Fear due to not understanding condition ,Frustration at lack of cure ,Treatment feels futile ,Hope for good keratoplasty recovery ,Hopeful for cure ,Initial misconception treatment is simple ,Fear at initial diagnosis ,Misconception of prognosis ,Misconception that condition could be cured with medication ,No concerns over treatment ,Misconception spectacles alone could fix issue ,Patient had not heard of keratoconus before diagnosis ,Patient not sure if condition can be cured ,Expect of curative treatment ,Poor ophthalmic health literacy ,Poor understanding of condition at young age ,Reassurance by treatment ,Researching own condition ,Researching treatment options ,Sadness at lack of cure ,Stoicism in the face of no treatment available ,Upset when thinking about treatment ,Wanting more advanced treatment options ,Wish for a cure ,Worry about progression ,Worry that keratoconus will affect cataract operation ,Appointment wait times are shorter in private clinics ,Appointment waiting rooms have frustrated staff and patients ,Appointments are frequent and frustrating ,Appointments are inconvenient ,Appointments are given at short notice ,Appointments are stressful ,Appointments feel like a waste of time ,Appointments feel pointless ,Appointments have a long waiting time ,Appointments have a long waitlist ,Appointments provide reassurance ,Appointments remind patient of their condition ,Appointments require long distance travel ,Appointments take a long time ,Frustration at needing to spend time for appointment ,Incorrect diagnosis from optometrist ,False hope given by referral ,Time cost of managing condition ,Difference between public and private health systems ,Difficult transitioning from public to private ,Frustration with public hospital ,Having to go to the hospital at a young age feels unfair ,Hospital environment is depressing ,Joining public system takes a long time ,Navigating public and private systems is confusing ,Nervousness visiting hospital ,Positive Experience with Hospital ,Private treatment is faster than public ,Positive experience with private treatment ,Public treatment surprisingly good ,Negative experience with public treatment ,Sadness at hospital experience ,Shock from seeing other patients at hospital ,Condition is diagnosed incidentally ,Condition is missed by optometrist ,Delayed diagnosis ,Difference in opinion between health care practitioners ,Disagreement with doctor over diagnosis ,Disagreement with practitioner if surgery is needed ,Doctors explain condition well ,Frustration at optometrist for creating false expectation that condition was treatable ,Frustration with doctor ,Frustration with not being taken seriously ,Inconsistent spectacle refraction results ,Consenting for surgery is scary ,Needing different practitioners for different aspects of treatment ,Negative interaction with treating doctor ,Nervous that junior doctor is performing treatment ,Ophthalmologist is waste of time as they cannot offer cure ,Optometrist equipment is not advanced ,Patient does not understand condition initially ,Patient does not want to discuss emotional distress with doctors ,Patient feeling powerless and frustrated at lack of explanation over management ,Patient happy to see specialist privately for post-op care ,Patient needs second opinion ,Positive experience with doctor ,Practitioner did not address patient's emotional distress ,Practitioner did not adequately explain treatment plan ,Practitioner did not mention keratoconus ,Practitioner needing second opinion ,Practitioner unsure of suitability for CXL ,Practitioner using confusing jargon ,Referral from optometrist was a waste of time ,Sudden unexplained changes to management ,Treated unprofessionally ,Carrying lens solutions is annoying ,Cleaning process for contact lens is annoying ,Unable to wear contact lenses due to prescription ,Contact lens causes dry eyes ,Contact lens causes irritation ,Contact lens falls out of eye easily ,Contact lens is annoying ,Contact lens need maintenance of a supply of solutions ,Contact lens usage is scary as it may cause graft rejection ,Fear of contact lens breaking in eye ,Futility with fogged rigid lenses ,Must follow tight schedule due to condition ,Ophthalmologist recommended RGP lens ,Optometrist advised RGP will be uncomfortable ,Patient believes contact lens will be uncomfortable ,Patient believes contact lens will improve QoL ,Patient believes Contact lens will improve vision ,Patient believes Contact lens will not improve vision ,Patient believes RGP lens cleaning routine is worth the vision improvement ,Patient believes RGP lens halts progression ,Patient believes RGP lens will be heavy ,Patient believes RGP lens will help ,Patient does not know how to insert contact lens ,Patient does not like idea of putting contact lens in eye ,Patient does not like RGP lens ,Patient fears poor outcome with contact lens ,Patient is dependent on scleral lens ,Patient unsure if contact lens will help ,Patient wishes for more comfortable alternative to RGP lens ,Piggyback lens is adequate ,Piggyback lens is itchy ,Piggyback lens is uncomfortable ,RGP lens accessories difficult to obtain ,RGP lens are difficult and painful to insert ,RGP lens are lost easily ,RGP lens can only be worn for limited time ,RGP lens cannot be worn during sport ,RGP lens cannot be worn for extended periods ,RGP lens cannot be worn in dry environments ,RGP lens cannot be worn underwater ,RGP lens causes watery eyes ,RGP lens cleaning routine is difficult to adapt to ,RGP lens cleaning routine is inconvenient ,RGP lens cleaning routine is lengthy ,RGP lens delayed by needing to see ophthalmologist first for CXL ,RGP lens falls out and patient needs help from others ,RGP lens falls out of eye easily ,RGP lens feels like foreign object in eye ,RGP lens fitting is unsuccessful ,RGP lens fitting process is scary ,RGP lens give good vision ,RGP lens handling requires clean hands ,RGP lens has a gritty sensation ,RGP lens has a long waiting time ,RGP lens has foggy vision ,RGP lens has inconsistent vision ,RGP lens has minimal improvement in vision ,RGP lens inconvenient to insert both lenses ,RGP lens insertion requires space and resources ,RGP lens interferes with treatment drop schedule ,RGP lens is clearer than spectacles ,RGP lens is difficult to wear ,RGP lens is easily broken ,RGP lens is easily mispositioned ,RGP lens is expensive ,RGP lens is not common ,RGP lens is older technology ,RGP lens is uncomfortable ,RGP lens makes eyes sensitive to wind and dust ,RGP lens makes eyes water ,RGP lens needs proper facilities to insert and remove ,RGP lens not worn even when prescribed ,RGP lens previous bad experience makes patient hesitant to try again ,RGP lens refitting is inconvenient ,RGP lens removal gives relief ,RGP lens requires goggles for swimming ,RGP lens requires multiple visits to fit ,RGP lens requires time to adapt ,RGP lens solution must be stocked in multiple places ,RGP lens solutions interrupt travel ,RGP lens solutions must be carried around ,RGP lens solutions need to be carried on person ,RGP lens solutions need to be fresh ,RGP lens solutions need to be stockpiled ,RGP lens wear is inconvenient for travelling ,RGP lens wear is riskier than glasses ,RGP lens wear limits time outside ,RGP lens will be expensive ,RGP wear feels unnatural ,Rigid lens can break in the eye ,Rigid lenses need to have their position in the eye adjusted ,Running out of contact lens solution is panic inducing ,Scleral lens cleaning routine is inconvenient ,Scleral lens does not fully correct vision ,Scleral lens enables daily functioning ,Scleral lens enables normal life ,Scleral lens gives clear vision ,Scleral lens has inconsistent vision ,Scleral lens has more stable vision than RGP ,Scleral lens has worse vision than RGP lens ,Scleral lens is better than RGP lens ,Scleral lens is expensive ,Scleral lens is more comfortable than RGP lens ,Scleral lens positive experience ,Scleral lens requires good practitioner ,Soft contact lens does not fully correct vision ,Soft contact lens feels like a foreign object in eye ,Sunglasses for protection against wind when wearing contact lenses ,Drops are annoying ,Drops are difficult to administer ,Drops are effective ,Drops are frustrating to carry around ,Drops are inconvenient ,Drops are not effective ,Drops are scary to use long term ,Drops bottle can injure eye ,Drops effect does not last ,Drops need to be carried on person ,Drops prevent eye rubbing ,Drops require multiple daily doses ,Drops require sleeping habit adjustments ,Patient is reliant on drops ,Antihistamine tablets have no effect ,Panadol provides no relief to headache ,Patient is not suitable for LASIK ,Reliance on antihistamine tablets for itchy eyes ,Taking vitamin supplements for vision ,Treatment is delayed due to cost ,Patient does not want to wear spectacles ,Patient is reliant on spectacles ,Practitioner advises spectacles will not help ,Spectacles are annoying ,Spectacles are effective ,Spectacles are only worn when needed ,Spectacles are uncomfortable ,Spectacles become ineffective due to progression ,Spectacles can be damaged ,Spectacles cause a headache ,Spectacles cause distorted vision ,Spectacles cause eye strain ,Spectacles cause glare ,Spectacles do not fully address symptoms ,Spectacles have a limit to their prescription ,Spectacles must be frequently changed ,Spectacles need replacing every few years ,Spectacles prescription changes after stitch removal ,Spectacles reduce confidence ,Spectacles restrict peripheral vision ,Spectacles are a part of patient's self-image ,Concern about need for repeat CXL ,Concern about public vs private CXL ,Concern over poor post-op outcome ,CXL costs the same public or private ,CXL delayed due to COVID19 ,CXL does not improve vision ,CXL experience is awful ,CXL is a scary experience ,CXL is delayed due to cost ,CXL is more painful than doctors advised ,CXL is not available publicly ,CXL is painful ,CXL is preventative not curative ,CXL is successful ,CXL needs booked accommodation for recovery period ,CXL no public option available ,CXL not suitable for older patients ,CXL overall good experience ,CXL post-op black eye ,CXL post-op cannot open eye ,CXL post-op discomfort from face shield ,CXL post-op drops must be used at work ,CXL post-op facial swelling and bruises ,CXL post-op pain ,CXL post-op pain is worse than expected ,CXL post-op pain severe ,CXL post-op recovery anxiety ,CXL post-op recovery is painful ,CXL post-op recovery requires family support ,CXL post-op recovery unexpectedly long ,CXL provides reassurance ,CXL recovery period is isolating ,CXL reduces progression ,CXL requires monitoring for suitability ,CXL stabilizes condition ,CXL suitability criteria is poorly understood by optometrist ,CXL was not available publicly initially ,CXL worsens vision ,Fear of junior doctor performing CXL ,Fear of mistakes during CXL ,Fear of vision loss from CXL ,Feeling they are given no choice in having CXL ,Frustration at post-op recovery ,Given false hope they are eligible for CXL ,Missing the treatment age for CXL is sad ,Nervous before CXL procedure ,Patient too old for CXL ,Patient wants CXL ,Previous CXL makes patients less nervous ,Previous CXL makes patients more nervous ,Worry that progression will occur unless surgery performed ,Anger at being told no treatment possible ,Anxious until recovery period over ,Concern over needing future surgery ,Frustration at lack of suitability for LASIK ,Surgery has a long waitlist ,Surgery has a short waitlist period ,Surgery has worse outcomes for keratoconus ,Surgery induces nervousness ,Surgery is delayed due to keratoconus ,Surgery is experienced awake ,Surgery is less scary after the first time ,Surgery is less scary with more advanced treatment ,Surgery is prevention, not cure ,Surgery is scary to research ,Surgery post-op deterioration ,Surgery post-op photosensitivity ,Surgery post-op stitches ,Surgery recovery time is long ,Surgery times are not clear and this causes anxiety ,Surgery vision outcome is good ,Worry that post-op outcome cannot be guaranteed ,ICRS post-op pain ,ICRS post-op photosensitivity ,Keratoplasty doesn't improve vision ,Keratoplasty improves vision ,Keratoplasty post-op eye graft is delicate ,Keratoplasty post-op improvement in vision ,Keratoplasty post-op minimal impact on QoL ,Keratoplasty post-op recovery period is long ,Keratoplasty post-op stitch changes prescription ,Keratoplasty post-op stitch requiring hospital visit ,Keratoplasty stabilizes vision ,Keratoplasty treatment experience was ok ,Keratoplasty is inconvenient ,Keratoplasty post-op avoiding water ,Keratoplasty post-op disturbed sleep ,Keratoplasty post-op drops ,Keratoplasty post-op drops annoying ,Keratoplasty post-op drops burden ,Keratoplasty post-op eye shield ,Keratoplasty post-op recovery occurring during lockdown makes it easier ,Keratoplasty post-op sleeping position change ,Keratoplasty anxiety during operation ,Keratoplasty donor tissue carries emotional burden to patient ,Keratoplasty minimal pain during operation ,Keratoplasty must be scheduled around life ,Keratoplasty needs repeat surgery ,Keratoplasty patient disagrees with doctor and wants surgery ,Keratoplasty patient feels surgery was unsuccessful ,Keratoplasty patient shocked when told they need transplant ,Keratoplasty patient told urgently they need transplant ,Keratoplasty patient unsure of visual outcome of surgery ,Keratoplasty patient views recovery as uncomfortable ,Keratoplasty Positive Experience ,Keratoplasty post-op appearance changes ,Keratoplasty post-op appointments are annoying ,Keratoplasty post-op black eye ,Keratoplasty post-op care requirements ,Keratoplasty post-op difficulties with daily activities ,Keratoplasty post-op improvement in vision for reading ,Keratoplasty post-op symptoms minimal ,Keratoplasty scheduled means patient cannot update glasses ,Keratoplasty surprisingly good ,Keratoplasty viewed as a necessity ,Keratoplasty worry that poor outcome will result in career change ,Long waiting period for keratoplasty ,Wanting an alternative to keratoplasty ,Psychological impact from keratoplasty ,Fear of ocular trauma after surgery ,Keratoplasty difference in expectation of post-op vision between doctor and patient ,Keratoplasty does not meet patient's expectations ,Keratoplasty improves outlook on condition ,Keratoplasty is a future consideration ,Keratoplasty is exciting ,Keratoplasty patient feels bad doctors do not want to perform surgery ,Keratoplasty patient vision expectations not met ,Keratoplasty post-op anxiety over damaging graft ,Keratoplasty post-op result is frustrating ,Keratoplasty post-op worry of damaging graft ,Keratoplasty provides hope ,Keratoplasty treatment experience was weird ,Keratoplasty was traumatising ,Poor post-op vision is frustrating ,Wearing eye protection as paranoid about damaging graft ,Worry about keratoplasty post-op pain ,Worry about repeat keratoplasty ,Worry about repeat of poor outcome in second keratoplasty ,Worry before surgery ,Worry over graft rejection ,Keratoplasty feels successful ,Keratoplasty graft rejection ,Keratoplasty post-op discomfort ,Keratoplasty post-op infection ,Keratoplasty post-op leak and discomfort ,Keratoplasty post-op pain ,Keratoplasty post-op pain minimal ,Keratoplasty post-op perforation ,Keratoplasty post-op steroid causes cataracts ,Keratoplasty post-op stitch irritation ,Keratoplasty post-op worsening vision ,Keratoplasty repeat procedure has worse vision ,Sadness at needing to change sleeping habits post-op

**Theme II: Symptoms**

Accepting the condition makes tolerating symptoms easier ,Adapted to coping with condition ,Adjusting resolution on computer screen ,Needing high visibility markings on stairs ,Falling down stairs ,Annoyance at symptoms ,Assistive devices are inconvenient ,Associating eye rubbing with progression ,Blur is only noticed occasionally ,Blurred vision makes patient scared of falling ,Cannot apply makeup well ,Cannot clean as well ,Change in personality due to frustration with symptoms ,Closing effected eye to reduce symptoms ,Clumsiness due to poor vision ,Concern about future progression of blur ,Concern about laser skin treatment effect on eyes ,Concern about loss of vision ,Concern over foreign body injuring eye ,Concern over progression in good eye ,Concern over progression in worse eye ,Condition isn’t ideal, but tolerable ,Counselling self through condition ,Denies needing to increase font size ,Difficulty recognizing others ,Difficulty carrying vision aids ,Difficulty judging distances ,Difficulty living with symptoms ,Difficulty reading ,Difficulty reading clock ,Difficulty reading documents ,Difficulty reading fine print ,Difficulty reading is annoying ,Difficulty reading phone ,Difficulty reading phone is annoying ,Difficulty reading posters ,Difficulty recognizing objects ,Difficulty with eye makeup ,Difficulty with low contrast text ,Difficulty with poor vision when waking up at night, no glasses ,Difficulty with stairs ,Difficulty with using phone ,Dim room lighting to reduce symptoms ,Distracting oneself to cope from symptoms ,Falls due to poor vision ,Fear of blindness ,Feeling older due to symptoms and coping mechanism ,Feelings of futility with symptoms not improving ,Feels condition could be worse ,Frustration and awareness of poor vision ,Frustration at needing rest breaks ,Frustration at needing to increase font size ,Frustration at poor vision ,Frustration at symptom burden ,Frustration at life-long condition ,Glare is a reminder of condition ,Grateful symptoms are not worse ,Home environment must be kept clean to reduce symptoms ,Inconvenience with leaning in to screens ,Increasing font size makes patient feel older ,Limited options with eye makeup due to irritable eyes ,Mindfulness to cope with symptoms ,Minor inconvenience due to symptoms ,Monocular blur causes worry ,Monocular blur hides condition ,Must be careful with cleaning around eyes ,Need to walk closer to see things ,Needing assistance from others with stairs ,Needing large text for reading ,Needing to change sleeping habits to reduce progression of condition ,Needing to kneel down to read things ,Needing to look down at stairs due to poor peripheral vision ,Needing to look down due to glare from sun ,Needing to look down to navigate stairs ,Needing to rely on senses other than vision ,Not noticing obstacles due to poor vision ,Not recognizing seriousness of condition ,Not sure if symptoms can be coped with mentally ,Obstruction of vision from glare ,Patient associates eye rubbing with condition ,Patient believes condition is related to screen usage ,Patient believes dry eyes are not associated with condition ,Patient considers clear vision important ,Patient considers their affected eye useless ,Patient considers their current vision very bad ,Patient does not notice condition ,Symptoms of early condition not noticed ,No Progression ,Patient prefers indoor environments ,Condition impacts everything in life ,Feeling of losing QoL ,Wanting clear vision ,Wanting better vision in both eyes ,Poor hand eye coordination ,Poor visual perception ,Poor vision makes patient feel condition cannot be treated ,Preferring indoor environments to control symptoms ,Preferring side of body associated with better eye ,Rapidly worsening vision is scary ,Reduced awareness of surroundings ,Reduction in symptoms has large improvement in QoL ,Relying on better eye causes fatigue ,Relying on hearing instead of sight ,Restricting rubbing of eyes ,Rubbing eyes due to itching ,Rubbing eyes when using screens ,Sadness at symptom burden ,Sadness that blurred vision will never improve ,Seeing a beautician as unable to do makeup themselves ,Self-consciousness over appearance of eyes ,Self-reassurance, ignorance of condition ,Sleep position changes associated with keratoplasty are annoying ,Squinting for blurred vision ,Squinting for blurry vision does not fully help ,Squinting is annoying ,Stress from fear of future blindness ,Strong mental health reduces impact of condition ,Sunglasses needed for glare ,Sustaining injury due to poor vision ,Symptom burden causes feelings of suicidality ,Symptoms are a reminder of mortality ,Symptoms are at forefront of mind ,Symptoms are constant reminder for condition ,Symptoms are immediately noticeable on waking ,Symptoms are present every day ,Symptoms make patient feel hopelessness ,Symptoms make patient feel older ,Symptoms make patient want to close their eyes ,Symptoms progress unnoticed ,Trying to ignore symptoms ,Unhappy that symptoms will not improve ,Unaware of condition ,Unhappiness due to persistent blur ,Unhappy with monocular blur ,Using phone to compensate for blurry vision ,Using phone to help with vision ,Walking slower due to worse vision ,Wants better unaided vision ,Washing face with water due to blurry vision ,Watery eyes due to light sensitivity ,Eye infection ,Discomfort ,Dry Eyes ,Dry Eyes in Morning ,Stinging ,Eye Rubbing from itching ,Eye Strain ,Headache ,Increased sensitivity to dust ,Inflammation ,Irritation ,Itch ,Concern about progression from rubbing ,Pain ,Photophobia ,Sensitive Eyes ,Soreness ,Interrupted sleep ,Sudden changes to symptoms ,Screen induced headaches ,Watery eyes ,Atopy ,Allergies ,Crusting ,Discharge ,Fatigue ,Hay fever ,Redness ,Swollen eyes ,Age changes symptoms experienced ,Air conditioning worsens symptoms ,Night time worsens blur ,Bright day worsens fatigue ,Difficulty adapting to changing light levels ,Difficulty in bright settings ,Dry air worsens symptoms ,Dust in good eye leads to poor vision ,Dusty environments worsens fatigue ,Dusty environments cause pain ,Symptoms improve in different countries ,Night time does not impact symptoms ,Itching is worse outdoors ,Pollen worsens symptoms ,Seasons affect severity of symptoms ,Windy environments worsen blur ,Windy environments trigger symptoms ,Windy environments cause discomfort ,Cold weather worsens symptoms ,Fatigue ,Sleeping incorrectly worsens blur ,Blur when closing good eye ,Stable vision ,Worsening vision ,Blurry vision ,Blurry distance vision ,Facial Recognition Difficulty ,Blurry monocular vision ,Blurry near vision ,Variable blurring of vision ,Head turn worsens blur ,Doesn't trust worse eye ,Blur is assumed as tiredness ,Worsening monocular blur ,Not noticing symptoms at young age ,Cloudy vision ,Blur varies with dry eyes ,Blur without glasses ,Double Vision ,Focusing difficulties ,Haloes around lights ,Loss of peripheral vision ,Worse night vision ,Poor depth perception ,Poor object recognition ,Rapid decline in vision ,Squinting ,Starburst of lights ,Symptoms insidiously progressed ,

**Theme III: Career**

Adopting abnormal head posture to read board at school ,Blur after extended near work ,Cannot read board in class ,Cannot read for extended periods of time ,Cannot read lecture notes ,Difficulty with research work in university ,Failing class due to keratoconus ,Having to sit closer to whiteboard at school ,Headaches interrupting learning ,Reading class notes afterwards instead of participating ,Reduced academic performance ,Reliance on digital whiteboard ,Requires darker writing on whiteboard to see ,Conflict with teachers due to using phone in class ,Stress from condition during school examination ,Stress from impacted education ,Teachers are understanding ,Teachers do not understand condition ,Teachers notice poor vision ,Deferred university semester ,Falling behind in education ,Giving up due to frustration from condition ,Interruptions to education due to condition ,Missing parts of lectures in university ,Reduced participation in schoolwork ,Requiring more breaks during study ,Stopping studies due to the condition ,Time taken off school ,Annual leave used for treatment recovery ,Being supported by workplace ,Being viewed as a liability at work ,Colleagues notice the keratoconus ,Contact lens solutions must be carried on person ,Difficulty using rigid contact lens in work environment ,Dry eyes when working in refrigerated area ,Dryness from using computer at work ,Eyestrain at work ,Feels incompetent at work ,Forgetting sunglasses impacts work comfort ,Frustration at reading numbers at work ,Frustration at using screen at work ,Having to hide their issues at work ,Inconvenience of taking medication during work ,Keratoconus impacts confidence in high stakes work ,Limited development of friendships at work ,Needing to lie for work ,Needing to take time off work ,Outdoor work leads to eye irritation ,Pain with using computer at work ,Patient Experience - Support from Work ,Patient is upset at stopping work ,Patient misses old career ,Pretending that they can perform tasks at work ,Reduced confidence in work environments ,Reduced safety at work due to the condition ,Rest breaks at work from screen ,Sadness at not being able to follow career goals ,Self-conscious at work ,Self-deprecation about condition at work or school ,Social work environment is uncomfortable ,Viewed by supervisors as underperforming at work ,Wanting to lie about condition ,Work is a distraction from the condition ,Working from home is more convenient for post-op recovery ,Cannot pass work standards for role ,Concern over career prospects ,Difficulty applying for jobs ,Discrimination in work environment due to condition ,Downgrading in careers ,Early understanding of limited career options due to vision ,Employers do not understand keratoconus ,Employers have not heard of keratoconus ,Fired due to keratoconus ,Impacted career opportunities ,Limited career progression ,Limited choice of career ,Needing to change careers ,Patient believes they will not be able to pass job test ,Pursuing an alternative career ,Unable to continue career ,Asking a colleague to read for them ,Avoiding computer usage at work ,Cannot avoid difficult tasks at work ,Difficulty in tight spaces due to being unable to use good eye ,Difficulty reading customer's facial expressions ,Difficulty reading letters at work ,Difficulty seeing text on computer ,Difficulty sighting objects at work ,Difficulty with detailed tasks at work ,Difficulty with using screens at work ,Given tasks at work suitable to poor vision ,Impacted performance due to difficulty judging distance ,Incorrect work at computer due to condition ,Increased contrast on screens ,Increased font size for devices ,Increased zoom for screen ,Increasing font size decreases content available on screen ,Increasing screen brightness ,Initial difficulty navigating new work environment ,Jobs with more screen usage are difficult ,Making mistakes at work ,Minimizing time on computer at work ,Needing brighter lighting at work to perform tasks ,Needing to change the types of tasks at work ,Needing to move closer to screens ,Needing to move closer to see objects ,Needing to stop work due to treatment ,Poor quality work due to condition ,Poorer performance at work ,Reduced concentration at work due to teary vision ,Reduced efficiency on computer at work ,Reliant on glasses for work ,Time wasted at work due to condition ,Unable to pass medical test for work ,Variable performance at work due to variable condition ,Variable work performance depending on vision ,Minimal impact on work from condition ,No impact on work from condition ,

**Theme IV: Enjoyment**

Denies impact on hobbies ,Limited ability to pursue interests ,Difficulty using assistive devices ,Cannot play sports with contact lens ,Cannot read for long before blur ,Cannot read sheet music anymore ,Cannot recognize teammates ,Cannot see opponents body ,Cannot see the ball in sports ,Cannot spend as long on hobbies due to symptoms ,Difficulty catching due to poor depth perception ,Difficulty reading menu at a restaurant ,Difficulty reading social media ,Difficulty with television subtitles ,Difficulty with weaker vision in one eye ,Difficulty editing photos ,Headaches while drawing and painting ,Bad experience using contact lens for sport ,Needing prescription goggles for sport ,Needing to pause television to read ,Needs to rely on glasses to see travel documents ,Passing the ball to the wrong player in soccer ,Poor performance due to vision ,Reduced ability in video games ,Reduced performance due to needing to play without vision aids ,Reduced peripheral vision due to glasses in sport ,Researching how to still enjoy hobbies ,RGP lens cannot be worn during sport ,Team doesn't accommodate for visual issue in sport ,Television difficult to see ,Television must be paused to read details ,Television must be squinted at ,Unable to capture happy memories ,Worsening performance in sport ,Limited confidence ,Fear of diving with contact lenses ,Fear of hiking ,Fear of lens falling out of eye during swimming ,Fear of swimming at beach ,Fear that hobbies will be restricted by reduced vision ,Needing supervision while fishing ,Others notice reduced ability ,Panic due to delays while travelling ,Reduced confidence in performance in sport ,Reduced confidence in video games ,Wanting supervision due to poor vision ,Worry of loss of ability to perform hobbies ,Limited participation due to condition ,Advised not to swim by doctors ,Avoiding parks and gardens due to pollen sensitivity ,Cannot dive with contact lens in eye ,Cannot obtain license to pursue aviation hobby ,Cannot take naps until lens is out of eye ,Difficulty driving to dancing classes ,No longer buying magazines due to poor vision ,Not dining out due to limited social life ,Not wanting to visit restaurants due to sensitive eyes ,Patient advised not to swim with contact lenses ,Poor night vision restricts nighttime outings ,Poor unaided vision restricts night time activities ,Reduced participation due to reduced confidence ,Restricting overseas travel due to fear of needing emergency treatment ,Restricting travel due to limited income from condition ,Restricting travel to certain countries due to pollution ,Sadness at stopping hobbies ,Stopping hobbies ,Stopping hobbies which require screentime ,Stopping video games due to eye rubbing ,Wants to continue hobbies again ,Limited pleasure ,Cannot enjoy 3D movies ,Blurry vision makes diving not enjoyable ,Cannot enjoy sightseeing while travelling ,Cannot enjoy social element of gym ,Concerts are not engaging ,Discomfort during air flights due to contact lenses ,Embarrassment from condition ,Eyes always closed during photos at social events ,Having glasses broken while playing sport ,Hobbies are a distraction from the condition ,Inconvenience of soft contact lens solutions during travelling ,Irritation from stadium lights ,Loss of passion for sport ,Missing a visual element of enjoyment ,Missing out on experiencing life ,Needing goggles to swim feels like a headache ,Poor vision limits ability to enjoy art ,Reduction in satisfaction from hobbies ,Relying on recordings instead of living in the moment ,Restricting overseas travel due to appointments ,Sadness at not being able to see kids succeed ,Embarrassment from protective goggles ,

**Theme V: Relationships**

Asking others to order food ,Avoiding eye contact due to self-consciousness ,Unhappiness from comparison with non-affected people ,Feelings of abnormality from condition ,Feelings of loneliness from condition ,Coping with self-deprecation ,Difficulty fitting in socially due to vision ,Discussing condition openly helps ,Disregarding other's opinions helps ,Patient feels their concerns aren't valid ,Futility from trying to make others understand the condition ,Hiding corneal graft from others with glasses ,Experiencing isolation ,Anger from lost self-confidence ,Not discussing condition ,Not wanting to be seen ,Not wanting to discuss condition with others ,Preferring familiar environments ,Self-conscious over appearance ,Worried other commuters will stare ,Worry that others notice red eyes ,Using support groups ,Wanting independence ,Wanting to leave social events early ,Wearing glasses reduces confidence when younger ,Denies impact on relationship ,Burdening family ,Concern condition is hereditary ,Difficulty spending time with family ,Unable to see kids succeed ,Support from family ,Needing understanding from family ,Needing respect from family ,Needing support from family ,Accidentally ignoring friends ,Banter with friends as a coping mechanism ,Burdening friends ,Difficulty socializing with friends ,Disengaging with friends ,Family and friends are a distraction from the condition ,Limited activities with friends ,Limited time with friends ,Others are hesitant to help patient with their makeup ,Needing respect from friends ,Needing understanding from friends ,Needing support from friends ,Appearing clumsy in front of others ,Awkward interactions at work ,Awkwardness with others ,Being judged by others ,Being seen as different ,Cannot relate to others with clear vision ,Experiencing Discrimination ,Feeling Different ,Inconveniencing others ,Isolation ,Lack of understanding ,Needing help from others ,Online support groups ,Disappointing teammates ,Avoiding Relationships ,Burdening partner ,Conflict with partner ,Difficulty dating ,Feelings of guilt due to relying on partner ,Difficulty planning future with partner ,Needing to explain condition to partners ,Preferring evening dates so eyes are less noticeable ,Work ,Asking someone to read for them at work ,Work banter to cope with condition ,Prioritizing friends despite blurred vision ,Difficulty socializing after work ,Discussing condition is positive ,Not developing friendships ,

**Theme VI: Driving**

Difficulty from poor depth perception ,Difficulty from blur ,Difficulty driving ,Difficulty reading maps ,Difficulty reading signs ,Difficulty reading speedometer ,Difficulty parking ,Difficulty seeing obstacles ,Spectacles are needed for driving ,Public transport is inefficient ,Public transport is worse with eye irritation ,Taking the wrong bus due to poor vision ,Difficulty reading public transport timetables ,Dust in eye interrupts bike riding ,Needing to use public transport ,Public transport is unreliable ,Riding a bike due to being unable to drive ,Difficulty driving with glare ,Difficulty driving in new areas ,Difficulty driving at night ,Difficulty driving in rain ,Difficulty driving at twilight ,Feeling like a burden on friends needing to drive them ,Concern about driving in the future ,Discomfort driving ,Concerned about their family and driving ,Fear of crashing their car ,Feeling unsafe while driving ,Feeling annoyed at poor vision while driving ,Sadness due to being unable to drive ,Avoiding highways while driving ,Being told not to drive by others ,Concerns and issues with driving license ,Contact lenses can interrupt driving ,Counting traffic lights while driving as they cannot read street signs ,Difficulty learning how to drive ,Difficulty with other lights ,Denies issues with driving license ,Feels night time driving is ok ,Feels driving is a positive experience ,Experiencing a car crash ,Fatigue while driving ,Fear over losing driver’s license ,Feeling like their mobility is restricted ,Needing sunglasses to drive ,Needing to be more careful while driving ,Needing to pullover due to poor vision ,Needing to take rest breaks while driving ,Needs glasses while driving ,Not looking at lights while driving ,Poor vision is a distraction while driving ,Relying on others to drive ,Using high beams while driving ,Driving is important for independence ,Difficulty driving in windy days ,

**Theme VII: Finances**

Public health system reduces costs ,Eyedrops are expensive ,Spectacles are expensive ,Replacement pairs of spectacles are expensive ,Spectacles are expensive ,Private consults are expensive ,Rigid lenses are expensive ,High costs are frustrating ,Contact lenses are expensive ,Losing rigid lenses is expensive ,Ongoing cost of contact lens solutions ,Ongoing cost of updating contact lens ,Rigid lenses expensive relative to student income ,Rigid lenses prohibitively expensive ,Scleral lenses are expensive ,Paying for vitamin supplements for condition ,Surgery is expensive ,Seeing private doctor is expensive ,Empathy for poorer keratoconus patients ,Frustration at repeat cost of medication ,High cost given life circumstances ,Needing to save for treatment ,Choosing between other necessities and treatment ,Poor vision improvement for money spent on treatment ,Money wasted on concert ,Financial strain on family ,Financial support from family ,Difficulty accessing welfare ,Inability to work ,Needing income from disability support ,Income increased by scleral lenses ,Income limited by job opportunities ,Income lost from time off work ,Cost of accommodation for treatment ,Cost of beauticians for makeup ,Increased life insurance premiums ,Cost of transport services due to inability to drive ,Cost of travel for treatment ,Cost of treatment for injuries related to keratoconus ,
